# Erroneous Causes of Point-of-Care Glucose Readings

**DOI:** 10.7759/cureus.36356

**Published:** 2023-03-19

**Authors:** Joseph Majewski, Zachary Risler, Kanika Gupta

**Affiliations:** 1 Emergency Medicine, Nazareth Hospital, Philadelphia, USA

**Keywords:** glucose, emergency medicine resident, knowledge of hypoglycemia, hyperglycemia, blood glucose monitoring

## Abstract

Recognizing and treating reversible causes of lethargy and altered mental status is crucial for emergency department physicians. One such tool that can quickly help guide resuscitation and a patient’s workup is a point-of-care glucose reading. This simple test is performed routinely; however, how much thought is given to the accuracy of these tests? What factors can alter these results? Here, we present a patient who was reported to be hyperglycemic in the field by emergency medical services (EMS) but was profoundly hypoglycemic during his workup in the emergency department. This case report highlights factors that can cause false hyper- and hypoglycemic readings on point-of-care glucose meters.

## Introduction

Point-of-care glucose meters can be found in clinical and nonclinical settings. Nonmedical and medical individuals utilize these meters daily to dictate medical decisions. For instance, point-of-care glucose readings can dictate resuscitation efforts in advanced cardiovascular life support (ACLS) by ruling out hypoglycemia, a reversible cause of cardiac arrest. Patients presenting hypoglycemia will often present with fatigue, dizziness, syncope, tremors, weakness, or even in a coma, which can be an imitator of strokes. Even with greatly reliable tools, errors can still occur. Human error, environmental factors, medications, and physiological factors are some examples. These inaccurate readings can largely alter the workup of a patient in the emergency department, and one such case occurred due to human error.

## Case presentation

The patient is a 55-year-old male with a past medical history of insulin-dependent diabetes mellitus who presented to the emergency department through emergency medical services (EMS). According to the rescue, the patient was found lethargic on the side of the road with a bag of candy in hand. In the field, blood sugar was reported to be high on the point-of-care glucose meter. Full history of presenting illness and a review of symptoms could not be obtained due to the patient's condition. On arrival, the patient's temperature was 36.3°C, heart rate was 93 bpm, respiratory rate was 18 breaths per minute, blood pressure was 135/77, and pulse ox on room air was 94%. On exam, the patient was alert and oriented (A&O)x0, unable to follow commands, had mild aphasia, regular pulse rate and rhythm, clear lungs to auscultation, and non-distended/non-tender abdomen, and his hands were covered in chocolate. The patients' hands were wiped with alcohol wipes, and the point-of-care glucose reading was 25. Two IVs and 2 amps of 50% dextrose (D50) were administered immediately. The patient's blood glucose was corrected to 231.

## Discussion

The basic principle of glucose meters is to take a drop of blood from the patient's finger and apply it to a single-use plastic test strip. These test strips contain enzymes and electrodes that will react with the glucose and cause an electrical signal that the meter will interpret.

Though point-of-care glucose meters are simple to use, basic principles such as cleaning the skin can greatly alter the readings. Glucose meters use tiny amounts of blood; hence, small contaminates like sugar products from fruits or candy as in our patient can affect the readings and change the course of treatments. The storage of test strips can also change the reliability of measurements. Storage of the meter and test strips together can cause mechanical stress to the electrode and substrate leading to hyper/hypoglycemic readings [1]. Proper patient education on hand hygiene and storage can help achieve proper results.

Environmental factors such as temperature can alter glucose meter readings. Modern glucose meters have built-in temperature sensors that will auto-correct measurements [1]. However, the temperature between the meter and the strip can differ leading to erroneous readings. Altitudes much greater than 2000 m have been shown to overestimate and underestimate readings [2]. One study has shown that glucometers underestimate glucose levels by 1%-2% for every 300 m of elevation [3]. This is believed to be caused by glucose oxygenase, which is dependent on oxygen for its reaction [3]. With an increase in altitude, there is a decrease in the partial pressure of oxygen. This decrease of dissolved oxygen in the blood and air can interfere with the reaction within the test strips, which ultimately decreases the electrical signal that the glucose meter interprets.

Highly elevated levels of acetaminophen as seen in overdoses can inaccurately alter the electrochemical system between the test strip and the meter leading to inaccurate readings. A case report of a 25-year-old female who overdosed on acetaminophen and diphenhydramine was found to be minimally responsive, so a point-of-care glucose check was conducted five times with five different meters [4]. Each glucose meter read as an error. Laboratory analysis concluded a glucose level of 180 mg/dl [4]. Acetaminophen can diffuse across the test strip electrode surface, oxidizing it. This leads to an interfering current that can cause inaccurate readings [4,5]. Icodextrin, a component of dialysis fluid, is metabolized into the disaccharide maltose [1,6]. Maltose is composed of two units of glucose. This sugar byproduct can be metabolized by the test strip enzymes into glucose, leading to falsely elevated blood sugar levels in dialysis patients.

Physiological factors including reduced peripheral blood perfusion in critically ill patients have been shown to cause false readings of hypoglycemia. These events were more common in devices that used glucose oxidase-based assays as oxygen is an important electron acceptor [1]. Patients with increased triglycerides can have false readings of hypoglycemia due to the volume that the triglycerides account for in the capillary space [2]. Patients undergoing chemotherapy or radiation can have excessive uric acid levels from DNA breakdown. These high levels of uric acid can oxidize the electrode and falsely cause hyperglycemic readings [2].

An overview of factors that can lead to erroneous readings on point-of-care glucose meters is listed in Table 1.

**Table 1:** Factors affecting point-of-care glucose meters

Factors affecting point-of-care glucose meters	Examples
Human error	Improper hygiene, storage, and use
Environmental	Temperature and altitude
Medications	Acetaminophen and dialysis fluid
Physiological	Tissue perfusion, hypertriglyceridemia, and chemo/radiation

## Conclusions

Multiple factors can result in imprecise blood sugar measurements. These readings can greatly alter the workup of a patient and delay diagnosis and treatment. Simple steps such as proper handling of equipment, storage, and hygiene can help avoid errors in point-of-care glucose readings.
